# COMMUNITY PREVALENCE OF POSTTRAUMATIC STRESS DISORDER, VETERAN STATUS, AND FALLS IN MA, CT, AND RI

**DOI:** 10.1093/geroni/igae098.3430

**Published:** 2024-12-31

**Authors:** Josephine Boateng, Alexa Fleet, Megan Siebecker, Taylor Jansen, Elizabeth Dugan

**Affiliations:** University of Massachusetts Boston, Dorchester, Massachusetts, United States; University of Massachusetts Boston-- Gerontology, Boston, Massachusetts, United States; University of Massachusetts Boston, Boston, Massachusetts, United States; University of Massachusetts Boston, Boston, Massachusetts, United States; University of Massachusetts Boston, Boston, Massachusetts, United States

## Abstract

Post-traumatic stress disorder (PTSD) is a debilitating condition that is not well-understood in older people and often undetected. PTSD may arise from military service, falls, or other traumatic events. Understanding the prevalence of PTSD is important to identify service gaps. This study uses data from the Healthy Aging Data Report (healthyagingdatareports.org). Indicator data sources included: Medicare Beneficiary Summary Files (2014-2015 MA, 2016-2017 CT and RI), the American Community Survey (2012-2016 MA, CT, RI) and BRFSS (2012 and 2014 MA, 2012-2018 CT, 2012-2017 RI). Small area estimation techniques were used to calculate rates using Stata and ArcGIS was used for mapping and geographic analyses. Results showed that community rates for PTSD were: CT 1.2% (0.41-3.10%), RI 1.7% (0.91-4.59%), MA 1.8% (0.58%-5.71%). Massachusetts had both the widest range in PTSD community rates and the highest state rate. Military service: MA 18.8% (3.24-42.58%), CT 17.4%, (6.70-33.7%), and RI 19.0% (9.51-31.70%). Falls: MA 10.6% (6.47-18.18%), CT 26.3% (19.31-32.39%), and RI 26.3% (21.51-31.17%). Disparities in access to care or clinicians who could diagnose PTSD may contribute to some of the differences observed. Understanding these regional disparities would inform collaborative efforts from researchers, policymakers and stakeholders. Further research is warranted on this unexplored topic.

